# Public health round-up

**DOI:** 10.2471/BLT.25.010825

**Published:** 2025-08-01

**Suriname certified malaria-free**

On 30 June, Suriname became the first country in the Amazon region to receive malaria-free certification from the World Health Organization (WHO). This historic milestone follows nearly 70 years of commitment by the government and people of Suriname to eliminate the disease across its vast rainforests and diverse communities.

So far, a total of 46 countries and 1 territory have been certified as malaria-free by WHO, including 12 countries in the Region of the Americas.

**Figure:**
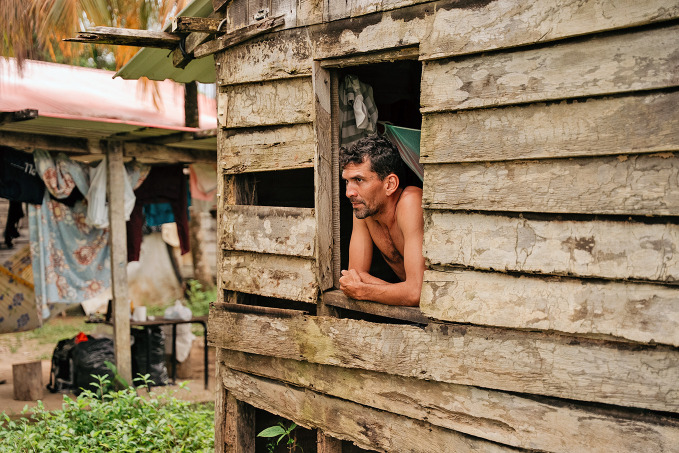
PAHO

## Trachoma elimination

Burundi and Senegal have been officially validated by the World Health Organization (WHO) as having eliminated trachoma as a public health problem, becoming the eighth and ninth countries in the WHO African Region, respectively, to do so. For both countries, this marks a significant public health triumph and reflects decades of commitment, community engagement and international collaboration. 

Trachoma is caused by the bacterium *Chlamydia trachomatis* and spreads through personal contact, contaminated surfaces and by flies that have been in contact with eye or nose discharge. Repeated infections can lead to scarring, in-turning of the eyelids and, ultimately, blindness. Globally, the disease remains endemic in many vulnerable communities where access to clean water and sanitation is limited.

In Burundi, trachoma was the first neglected tropical disease (NTD) to be eliminated. The country launched targeted efforts after baseline surveys in 2009–2010 confirmed endemicity in several health districts. With support from partners including Christoffel Blindenmission, the END Fund, and the International Trachoma Initiative, Burundi implemented interventions for 2.5 million people in 12 districts.

Senegal, which began nationwide mapping and interventions in 1998, reached 2.8 million people across 24 districts using the WHO-recommended SAFE strategy. Trachoma is the second NTD to be eliminated in Senegal, the country previously eliminated dracunculiasis (Guinea-worm disease) in 2004 and continues efforts to combat other NTDs.

WHO Director-General Tedros Adhanom Ghebreyesus praised both countries for their achievements, which underscore growing momentum in the global fight against NTDs. WHO continues to support health authorities in both countries to monitor communities in which trachoma was previously endemic to ensure there is no resurgence of the disease.

https://bit.ly/4o57xNd

https://bit.ly/4oaSZvK

## Mental health dashboard

WHO’s South-East Asia Region has launched a new regional dashboard on mental, neurological, substance use and self-harm conditions (MNSS) to help countries in the region strengthen data-driven planning, monitoring and evaluation of mental health services. The tool comes amid a growing mental health burden in the South-East Asian Region, where an estimated 289 million people live with MNSS conditions and nearly 208 000 people die by suicide each year.

The dashboard provides disaggregated data by country, gender, age group and condition, helping policy-makers and health professionals design more responsive interventions. It supports implementation of the WHO *South-East Asia mental health action plan 2023–2030* and aligns with commitments under the Paro Declaration on Universal Access to People-Centred Mental Health Care.

“This burden is often silent, but it is not abstract. It is a tangible experience lived by individuals, families and communities… our response must be equally tangible. It must be rooted in evidence and shaped by data,” said Dr Catharina Boehme, Officer-in-Charge, WHO South-East Asia Region.

WHO emphasizes that data must drive meaningful action to close mental health-care gaps and ensure resilient, compassionate systems for all.

https://bit.ly/4f7vDCN

https://bit.ly/4faSeyw

## Attack on WHO warehouse in Gaza 

WHO has condemned in the strongest terms a series of attacks on its facilities in Deir al Balah, Gaza, including the repeated targeting of a building housing WHO staff and the destruction of its main medical warehouse. The assaults, on 20 and 21 July, followed intensified hostilities and evacuation orders by Israeli military forces. Israeli military entered the premises, forcing women and children to evacuate on foot toward Al-Mawasi amid active conflict. Male staff and family members were handcuffed, stripped, interrogated on the spot, and screened at gunpoint. Two WHO staff and two family members were detained. Three were later released, while one staff member remains in detention. Thirty-two people, including women and children, were collected and evacuated to the WHO office in a high-risk mission, once access became possible. 

WHO’s operational capacity is now critically impaired, with most staff housing inaccessible and its warehouse damaged and looted. Medical supplies are nearly exhausted, severely limiting support to Gaza’s collapsing health system. WHO is urgently calling for the protection of its staff and premises, immediate humanitarian access, as well as a sustained and regular flow of medical supplies into Gaza.

With 88% of Gaza under evacuation orders or militarized zones, WHO warns there is no safe place to operate, underscoring that a ceasefire is not just necessary, it is long overdue. 

https://bit.ly/3TXDVUk

## Global childhood vaccination coverage

Global childhood immunization rates held steady in 2024, with 89% of infants, about 115 million, receiving at least one dose of the DTP (diphtheria, tetanus, pertussis) vaccine and 85% completing the full three-dose series, according to new data from WHO and the United Nations Children's Fund (UNICEF). While this marks modest progress, 14.3 million children still received no vaccines at all, and nearly 20 million missed at least one dose.

Low-income countries supported by Gavi, the Vaccine Alliance, showed encouraging improvements, reducing under-vaccination by about 650 000 children. However, progress remains deeply unequal. Conflict-affected and fragile states, home to 25% of the world's infants, now account for half of all unvaccinated children globally.

"Vaccines save lives, allowing individuals, families, communities, economies and nations to flourish," said Tedros Adhanom Ghebreyesus, WHO Director-General. "It's encouraging to see a continued increase in the number of children being vaccinated, although we still have a lot of work to do."

Persistent challenges, such as misinformation, shrinking health budgets and instability continue to threaten immunization gains, WHO and UNICEF warn. Both agencies call for urgent action to close equity gaps and prevent a reversal of decades of progress in protecting children from preventable diseases. 

https://bit.ly/4nXdPym

## Pandemic Agreement

WHO Member States convened the first meeting of the Intergovernmental Working Group (IGWG) on the WHO Pandemic Agreement from 9–10 July 2025, marking a critical step towards implementing this landmark global accord adopted at the Seventy-eighth World Health Assembly. The IGWG’s primary task is to draft and negotiate an annex on Pathogen Access and Benefit Sharing (PABS), a system designed to ensure safe, transparent and equitable sharing of pathogen materials and genetic sequence data to strengthen pandemic prevention, preparedness and response. The final annex will be submitted to the World Health Assembly in 2026.

The IGWG also began work on procedural matters, including preparations for the Conference of the Parties to the Agreement and development of a Coordinating Financial Mechanism. The meeting established the group’s method of work, elected leadership and agreed on timelines and expert consultations.

“Through the WHO Pandemic Agreement, countries recognized that global collaboration and action, based on equity, are essential for protecting people from future pandemics,” said Ambassador Tovar da Silva Nunes of Brazil, IGWG co-chair. “Now, through the IGWG, countries are breathing life into the Agreement by establishing the way forward to implement its life-saving provisions.”

https://bit.ly/46tAP1U

## Raising health taxes

WHO launched the “3 by 35” Initiative, calling on governments to raise real prices on tobacco, alcohol and sugary drinks by at least 50% by 2035 through health taxes. The goal is to curb the rising toll of noncommunicable diseases (NCDs), which cause over 75% of global deaths, and to mobilize 1 trillion United States dollars (US$) in public revenue over the next decade. A 50% price hike on these products could prevent 50 million premature deaths over 50 years.

“Health taxes are one of the most efficient tools we have,” said Dr Jeremy Farrar, WHO Assistant Director-General for Health Promotion. “They cut the consumption of harmful products and create revenue governments can reinvest in health care, education and social protection.”

Many countries have already raised tobacco taxes with success, but harmful subsidies and tax loopholes remain common. WHO’s initiative will support countries with technical guidance, policy expertise, and partnerships to implement effective tax reforms and strengthen health systems through sustainable, domestic financing. 

https://bit.ly/44TUreo

**Cover photo**

A health worker screens a community resident for trachoma in Cantón El Rodeo, Tacuba district, El Salvador.

**Figure:**
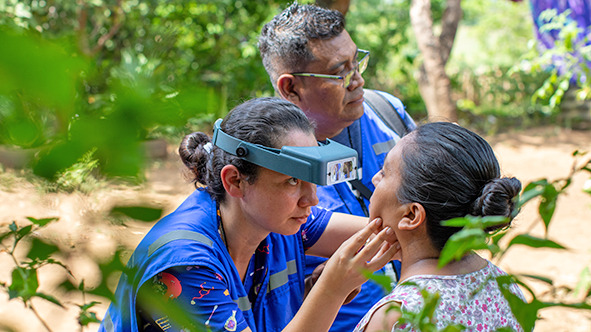
PAHO

**Looking ahead**

1–7 August. World Breastfeeding Week, global events. https://bit.ly/3Uwy5cC

17 September. World Patient Safety day, global events. https://bit.ly/4kPVpNb

22 September. Meeting of the Strategic Advisory Group of Experts on Immunization (SAGE), WHO Headquarters, Geneva. https://bit.ly/4f7O3nb

